# Antigen vehiculization particles based on the Z protein of Junin virus

**DOI:** 10.1186/1472-6750-12-80

**Published:** 2012-11-02

**Authors:** Cristina S Borio, Marcos F Bilen, Marcelo H Argüelles, Sandra E Goñi, Javier A Iserte, Graciela Glikmann, Mario E Lozano

**Affiliations:** 1 LIGBCM-AVEZ. Department of Science and Technology, Universidad Nacional de Quilmes, Bernal, Buenos Aires, Argentina; 2 LIGBCM-AVI. Department of Science and Technology, Universidad Nacional de Quilmes, Bernal, Buenos Aires, Argentina; 3 Laboratory of Immunology and Virology. Department of Science and Technology, Universidad Nacional de Quilmes, Roque Saenz Peña 352, Bernal, Buenos Aires, B1876BXD, Argentina

**Keywords:** Virus-like particles, Antigen delivery, Arenavirus, Z protein

## Abstract

**Background:**

Arenavirus matrix protein Z plays an important role in virus budding and is able to generate enveloped virus-like-particles (VLPs) in absence of any other viral proteins. In these VLPs, Z protein is associated to the plasma membrane inner surface by its myristoyl residue. Budding induction and vesicle formation properties can be exploited to generate enveloped VLPs platform. These structures can be designed to carry specific antigen in the inner side or on the surface of VLPs.

Vaccines based on VLPs are a highly effective type of subunit vaccines that mimic the overall structure of virus particles in absence of viral nucleic acid, being noninfectious.

In this work we assayed the capacity of Junin Z protein to produce VLPs carrying the green fluorescent protein (eGFP), as a model antigen.

**Results:**

In this report the Junin Z protein ability to produce VLPs from 293T cells and its capacity to deliver a specific antigen (eGFP) fused to Z was evaluated. Confocal microscopy showed a particular membrane bending in cells expressing Z and a spot welded distribution in the cytoplasm. VLPs were detected by TEM (transmission electron microscopy) and were purified from cell supernatant. The proteinase protection assay demonstrated the VLPs integrity and the absence of degradation of the fused antigen, thus indicating its internal localization. Finally, immunization of mice with purified VLPs produced high titres of anti-eGFP antibodies compared to the controls.

**Conclusions:**

It was proved that VLPs can be generated from cells transfected with a fusion Junin virus Z-eGFP protein in absence of any other viral protein, and the capacity of Z protein to support fusions at the C-terminal, without impairing its budding activity, allowing vehiculization of specific antigens into VLPs.

## Background

The Arenavirus Z protein is considered the matrix protein of this virus family [1]. It comprises three main regions that contain different domains: the Amino-end (Myristoylation Domain), the Core (RING Domain), and Carboxyl-end (Late Domain) [2]. Among the several functions described for this small protein (11 kDa) it can be highlighted its inhibitory effect on viral RNA replication and transcription through its interaction with the L protein [3,4] as well as its capability of interacting and recruiting the viral nucleoprotein [5,6]. Other interesting proposed properties may include: translation inhibition through interactions with cellular factors like the promyelocytic leukemia protein (PML) [7], the eukaryotic translation initiation factor eIF4E [8], and downregulation of IRF7 expression in arenavirus-infected plasmacytoid dendritic cells (pDCs) [9]. At the late steps of the infection cycle, Z is the driving force of arenaviral budding [10]. This activity is mainly conducted by two motifs found at the Z C-terminal domain, PTAP in the New World arenaviruses and/or PPPY in the Old World arenaviruses. These motifs, called late domains, mediate the interaction with cellular factors of the multivesicular body pathway (MVB) and drive the viral budding from the plasma membrane of arenaviruses and other viruses [9,11]. It has been previously reported that the expression of the Z protein alone is sufficient to induce the release of Z-containing enveloped particles in Lassa fever virus [10,12], Mopeia [6], Tacaribe and Junín [13,14]. A myristoylation in the glycine 2 residue is an important modification of Z. Mutations at this site completely abolish its capacity of driving the budding process, reflecting the importance of the interaction with membrane to promote budding [15,16]. For other matrix proteins, it has been reported that insertion of epitopes with a length of 20–30 amino acids still allows VLPs production and does not interfere with its budding activity [17].

Budding induction and vesicle formation properties can be exploited to generate enveloped VLPs. These VLPs structures can be designed to carry T or B cell epitopes which can be recognized by mature T and B lymphocytes from adaptive immune response [17]. Currently, many successful viral vaccines are predominantly based on live attenuated or inactivated viruses. The induced immune responses are similar to those from natural infections and often these vaccines are effective after a single dose. However, the reversion of attenuated viruses and incomplete inactivation of killed virus vaccines are the major manufacturing concerns. Subunit vaccines are safer than live attenuated and inactivated vaccines, but often single proteins, when expressed and purified in the absence of other viral components, are less immunogenic than those that are incorporated into infectious particles. In this sense, the vaccines based on VLPs are a highly effective type of subunit vaccines that mimic the overall structure of virus particles in absence of viral nucleic acid, being absolutely noninfectious [18].

In this work we assayed the capacity of the Junin Z protein as a VLP-forming protein carrying the green fluorescent protein (eGFP) as a model antigen. To accomplish this, we cloned and expressed in mammalian 293T cells the recombinant Z-eGFP from the Z protein of Junín Virus Candid#1. In addition, we demonstrated that the fusion of Z protein to a heterologous protein like eGFP, does not interfere with its budding capacity in mammal cells, evaluating the VLP generation by microscopic and immunologic methodologies. Furthermore, we produced and purified Z-eGFP derived VLPs to evaluate their ability to induce an immune response *in vivo*.

## Results

### Expression of Z-eGFP on 293T cells

**Figure 1:**
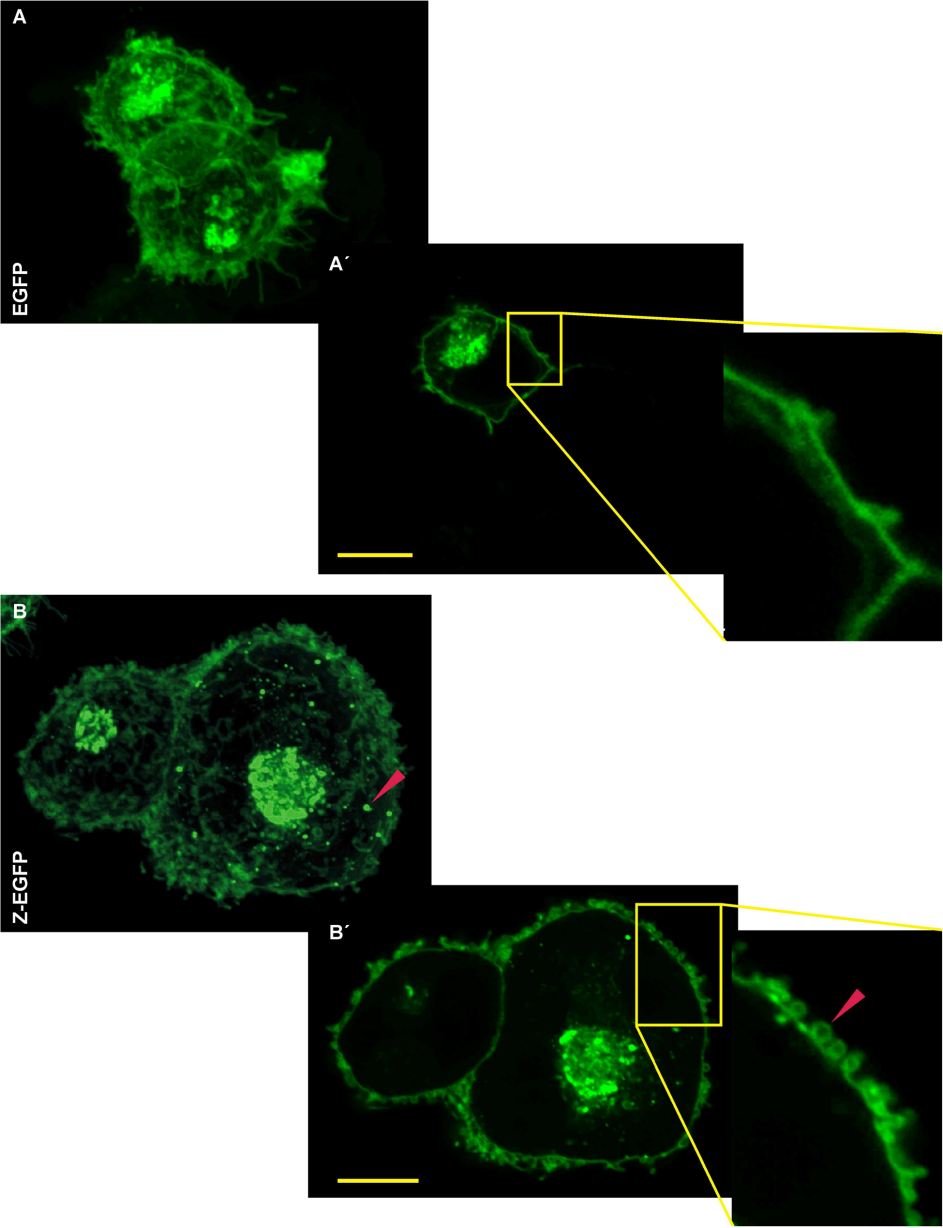
**Confocal fluorescence microscopy of 293T transient transfected cells. A**. Z-serie of 293T control cells transfected with peGFP-N3, followed by an optical section and digital zoom of the yellow rectangle. **A**´. Optical section. **B**. Z-serie of 293T cells transfected with pZ-eGFP, followed by an optical section and digital zoom. **B**´. Optical section. Red arrows indicate the membrane bending and the spot welded distribution. The bar in each figure represents 10 μm.

The pZ-eGFP plasmid was constructed based on the commercial vector pEGFP-N3 (Clontech) for mammalian cell expression of Z-eGFP protein under the regulation of CMV promoter. Transient transfection of 293T cells with this plasmid resulted in a high level expression of Z-eGFP protein (37.9 kDa). We analyzed the expression of both Z-eGFP and eGFP (26.9 kDa) proteins by confocal fluorescence microscopy and the distributions were similar in both cases, only with minor differences (Figure 1). In Z-eGFP transfected cells (Figure 1B) we observed a dotted pattern of fluorescence distributed around the cytoplasm (close to the plasma membrane) which was completely absent in control cells (Figure 1A). These patterns were clearly ascertained in optical sections (Figure 1B’ and Additional file 1: Figure S1A). The main difference between Z-eGFP and eGFP expression resides in the plasma membrane bending in Z-eGFP transfected cells. This phenomenon is clearly observed on the Z stack as well as in the optical sections (Figure 1B’) but it was also absent in the corresponding control cells (Figure 1A and 1A’).

### Purification of Z-eGFP virus like particles

**Figure 2:**
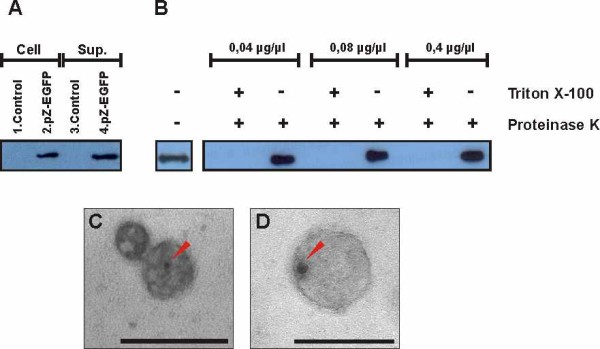
**Z-eGFP virus like particles detection and characterization. A**. Western Blot with anti-Z inmunoglobulines of Z-eGFP expression in 293T cells. Lane 1. 293T untransfected monolayer (control). 2. 293T monolayer transfected with pZ-eGFP. (Cell) Cell lysate 3. Pellet of the ultracentrifugated supernatant of untransfected 293T cells (control). 4. Pellet of the ultracentrifugated supernatant of 293T cells transfected with pZ-eGFP. (Sup) pelleted material from supernatant. **B**. Protease protection assay of Z-protein-containing particles in the supernatant of pZ-eGFP transfected 293T cells. Western Blot with anti-Z inmunoglobuline of the pellet of the ultracentrifugated supernatant of transfected cells, treated with proteinase K to final concentration of 0.04 μg/μl, 0.08 μg/μl and 0.4 μg/μl, with or without Triton X-100, or untreated (lane 1). **C** and **D**. Transmission electron microscopy with immunogold labelling of the pellet obtained by ultracentrifugation through a sucrose cushion of the supernatant of pZ-eGFP transfected cells. The red arrow indicates the gold labelling. The bar in each figure represents 100 nm.

In order to determine if the fluorescent signal corresponded to the full length Z-eGFP protein, the cell lysate fraction was analyzed by western blot and the Z protein was detected (Figure 2A), with a molecular weight of approximately 38 kDa, corresponding to the size of the fusion protein. To detect virus like particle formation, we analyzed the pellet after ultracentrifugation through a sucrose cushion of supernatants harvested from pZ-eGFP transfected cells and, as shown in Figure 2A, we localized the Z-eGFP in the pelleted material.

To analyze if Z-eGFP was included into organized structures resembling VLPs, the pellet obtained after ultracentrifugation was subjected to a protease protection assay (Figure 2B). In this assay, untreated samples were compared to samples treated either with proteinase K alone or with proteinase K in the presence of Triton X-100 to allow permeabilization. Treatment with proteinase K had no effect on the Z-eGFP protein, whereas the addition of Triton X-100 destroyed the lipid envelope and therefore enabled the degradation of Z-eGFP by proteinase K. These results clearly demonstrate that Z-eGFP is located in the inner region of VLPs and is preserved from degradation by the lipid membrane. Finally, this sample was analyzed by transmission electron microscopy with immunogold labeling (Figure 2C and D, and Additional file 2: Figure S2), which provided evidence for the presence of irregular electron dense structures of 40 to 150 nm of diameter coinciding with the expected appearance of VLPs labeled with gold particles. These particles were absent in the control group (data not shown). Furthermore, in Additional file 2: Figure S2 we observed a structure, compatible with membrane debris, containing Z-EGFP. Also in the TEM picture, unlabeled VLPs were observed, probably due to the sample preparation protocol, which lacks a permeabilization step.

### GFP-specific IgG antibody response

**Figure 3:**
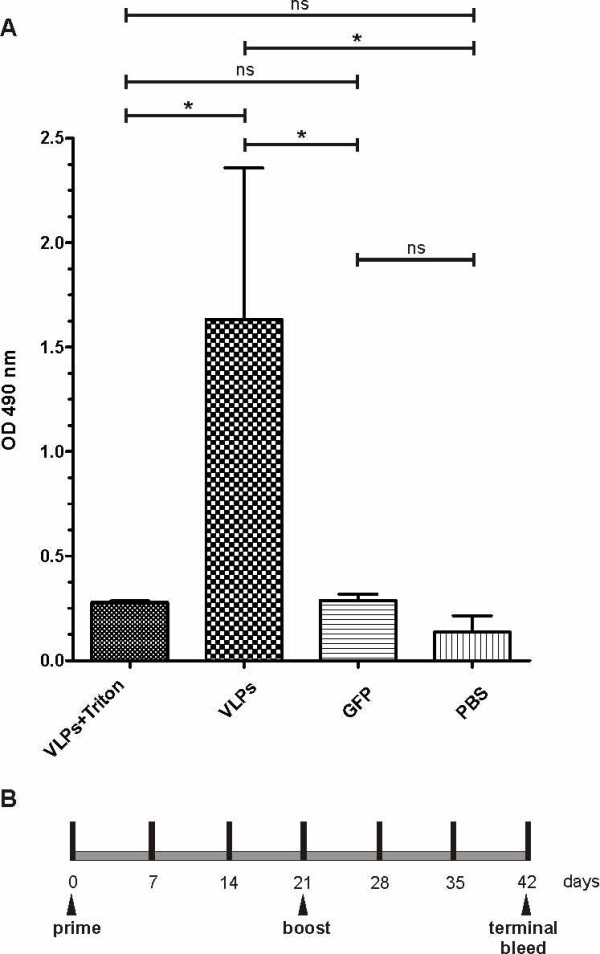
**GFP-specific total serum IgG antibody response. A**. Groups of Balb/C mice were intramuscularly immunized twice with Z-eGFP VLPs, Z-eGFP VLPs treated with Triton X-100, GFP and PBS (negative control). Blood samples collected at day 35 were 50-fold diluted and used to determine GFP-specific total IgG by ELISA. Optical densities (490 nm) are expressed as the arithmetic means plus standard error. ANOVA one-way analysis (Tukey's Multiple Comparison Test) of variance was used to determine statistical significance between different samples.*; P<0.05. ns; not significant (P>0.05). **B**. The immunization schedule used in this experiment.

To determine the humoral immune response to GFP, Balb/C mice were immunized with the previously purified Z-eGFP VLPs, in the absence of adjuvant. Total GFP-specific IgG antibodies were assayed by ELISA on GFP coated plates (Figure 3). After day 21 most of the mice belonging to VLPs immunized group had generated an immune response to GFP, while maximum titres were achieved by day 35 (1:1000 on average). The arithmetic means of each group at day 35 were plotted in Figure 3, where it was possible to evidence that the IgG response obtained by Z-eGFP VLPs was significantly more robust than the soluble antigen, or the Triton X-100 treated Z-eGFP VLPs.

## Discussion

In this study we analyzed the Junin Virus Z-eGFP fusion protein expression and, in more detail, its budding capacity on 293T cells, aiming to the development of a platform for antigen vehiculization.

First, we assayed the transient transfection of 293T cells with pZ-eGFP (Figure 1), where we detected high levels of Z-eGFP expression with a homogeneous distribution. However, the resolution achieved by confocal microscopy allowed us to detect some regions of concentrated protein which are located near the nucleus, where it would be expected to find the endoplasmic reticulum membrane system, and a spot welded distribution around the cytoplasm. These spots can be explained by the previously described interaction between Z and the PML protein. The PML forms nuclear bodies, and it has been shown that Z alone is sufficient to redistribute PML from a nuclear punctuate to a cytoplasmic punctuate pattern [7]. Also these agreggates could be late endosomal compartments such as multivesicular bodies (MVB) containing intraluminal vesicles derived from Z-eGFP, as it has been described for retroviruses [19]. These vesicles would then be transported to the plasma membrane, where the fusion of the MVB allowed the release of the intraluminal vesicles, in this case the Z-eGFP derived VLPs. This hypothesis of Z-eGFP associated to membrane vesicles is supported by the fact that arenavirus Z protein is myristoylated. The wild type distribution of Z protein is spot welded around the cytoplasm and homogeneously distributed across the plasma membrane. However, in studies with a mutant Z protein at the myristoylation essential amino acid (glycine 2) a diffused pattern mainly with a perinuclear distribution was shown [15]. The same was demonstrated for the gag protein of HIV [20]. Therefore it has been proved that Z localization is membrane associated [15], and it has been postulated that vesicles transport the protein from the place of synthesis and myristoylation, to the plasma membrane. However, Kentis *et al.*(2002) [21] described that bacterially expressed LCMV Z protein appeared to self assemble into ordered protein structures of 500 Å that approximate in size to the protein aggregates observed *in vivo*. These arrangements were obtained *in vitro* from purified Z protein expressed in a prokaryotic system where some posttranslational modifications, like myristoylation, do not occur.

On the other hand, the morphology of the membrane on cells transfected with pZ-eGFP clearly differed from control cells (eGFP). In the first case, it was possible to observe the bending of the plasma membrane or budding initiation regions, suggesting an intact budding activity of Z-eGFP protein. This membrane bending has also been observed using confocal fluorescence microscopy for other viruses like AcMNPV baculovirus [22] and MLV retrovirus [23].

Results depicted in Figure 2 showed that recombinant Z-eGFP protein was able to form vesicles at the plasma membrane in a similar way as Z protein alone, suggesting that the fusion of a 238 amino acid protein, like eGFP, did not impair the budding capacity of Z. It is a common feature of matrix polypeptides like HIV gag protein or influenza M1 protein to conserve their budding capability when they are fused to small epitopes [17]. However, it is not frequent that proteins mainly involved in budding activities can support the introduction of large proteins without having its budding capacity impaired. Capul and de la Torre (2008) [24] have recently described that the fusion of the small (185 amino acids) luciferase from *Gaussia princeps* to Arenavirus Z protein resulted in a quimeric protein that retained budding activity. Considering the budding capacity of quimeras containing Z from Junín virus and a heterologous protein (in this study, GFP), other proteins could be coupled to Z generating a tool for antigen delivery.

According to the electron microscopy images the VLPs produced by Junin virus Z protein in 293T cells have a relative size similar to the Junin virion (50 to 100 nm) (Figure 2C and D) as well as an irregular shape, which is consistent with observations made for other arenaviruses [12,25,26].

Some important features for an immunization delivery vehicle are a safe generation procedure in a low biosafety level lab and the protection of the internal molecules from degradation. According to this, VLPs lack of any replicative function and the results show that particles are closed and internal polypeptides (in this study, Z-eGFP) protected from extracellular proteases. In order to analyze the VLPs immunization capacity, an assay using Balb/C mice was performed. A satisfactory humoral immune response was induced against the eGFP contained within the VLPs. This response was detected within a few days after the booster immunization reaching a maximum title by day 35. However, in the same conditions, neither completely disrupted Z-eGFP VLPs nor purified eGFP immunized mice showed a detectable IgG response (Figure 3), suggesting a better performance for intact VLPs.

There are many reports showing that the particulate nature improves the immunogenicity of the proteins included in or delivered by VLPs [27,28]. So, it is not surprising that VLPs described here improved the response against the antigen contained within the particle. As mentioned before, an equal dose of purified soluble protein did not induce a detectable humoral response. Traditional VLPs as well as pathogens have highly repetitive surfaces and usually induce strong antibody responses because they can cross-link B cell receptors with high efficiency [29]. The vesicles generated here show an important difference with other VLP systems since the protein is contained mainly within the vesicle, as shown by the protease protection assay (Figure 2B). In order to induce production of antibodies, protein must be exposed so that it can be recognized by B cells and this might happen by partial disruption of VLP membrane with exposition of antigen and direct stimulation of B cells. Although the size of VLPs (50–100 nm) would allow their access to lymphatic vessels and their transport directly to draining lymph nodes [28,30] we can not exclude the possibility that cell-associated transport of native protein might be involved in activation of B cells as well. This phenomenon, that involves internalization and recycling of native antigen to cell surface, has been described for macrophages associated to the subcapsular sinus [31-33], dendritic cells [34-36] and follicular dendritic cells [37]. This mechanism would be important in dendritic cells, which have both non-degradative and degradative antigen uptake pathways, allowing them not only to prime T cells but also to present native antigen to B cells. Some authors have demonstrated that this mechanism is dependent of interaction of immune complexes with inhibitory Fc receptors [38] but a recent publication from Le Roux *et al*. demonstrates that DCs have the ability to regurgitate unprocessed antigen which poses another B-cell activation mechanism [39].

Any of these possibilities or more probably a combination of several of them would allow inducing an antibody response against eGFP, despite the fact that the protein is contained within the VLPs (Figure 3). Similar results were obtained by Cubas *et al.* (2009) [40] after immunizing mice with SHIV VLPs (SIV Gag + HIV SF162 Env), since they observed an antibody response both to external envelope protein (Env) and to the internal gag protein.

Although the immunological response induced needs further characterization the results suggest that this system might be used as a platform to deliver foraneous antigens or even chimeric heterologous antigens, which can induce an antibody response even in the absence of any exogenous adjuvant.

## Conclusions

In summary, the results obtained here indicate that Z protein from Junin virus can support fusions at the C-terminal without impairing its budding activity. This is suitable for the production of VLPs and could be used for vehiculization of heterologous proteins from other pathogens acting as promising immune stimulators.

## Methods

### Cell culture

For the transient expression of recombinant Z protein we used human embryonic kidney cells transformed with adenovirus type 5 DNA, designated 293T [41]. Cells were grown in RPMI 1640 (Invitrogen) supplemented with 10% fetal bovine serum at 37°C in a 5% CO_2_ atmosphere.

### Transfection of 293T cells

Cell monolayers (80% confluence) were washed with PBS and transfected with the plasmid using PolyFect transfection reagent (Qiagen) following the manufacter instructions. The ratio of DNA (μg) to PolyFect (μl) was 1:10 for both 30 mm dishes (2 μg of DNA) and 60 mm dishes (8 μg of DNA). In all cases the DNA was previously purified with Qiagen Plasmid Mini kit. The samples were analyzed by Western blot and Fluorescence Microscopy as described below, 48 and 72 h post-transfection.

### Fluorescence microscopy

Transfected 293T cells with either pZ-eGFP or peGFP-N3 were grown on glass cover slides as indicated above. After 48 h, cells were fixed with 4% (wt/vol) formaldehyde in PBS for 15 min at room temperature. Images were captured using a confocal microscope Olympus FluoView FV1000 at 600x magnification, and analyzed using Olympus FluoView 2.0 software.

### Western blot

The samples were separated by SDS-PAGE (12-15% polyacrylamide gel) and blotted onto nitrocellulose membranes (Hybond P+, Amersham Pharmacia) in Tris-glycine buffer containing 20% (v/v) methanol. To avoid non-specific binding of the antibodies, the membranes were blocked by incubating with 5% (w/v) skimmed powder milk in PBS for 2 h at 37°C. For the primary antibody incubation the blocked membrane was probed with a 1/1,000 dilution of a polyclonal antiserum specific for the Z protein on PBS 2% casein at 37°C for 1 h, followed by a horse radish peroxidase conjugated goat anti-rabbit IgG (Santa Cruz Biotechnology) incubation, diluted 1/10,000 on PBS 0.1% Tween-20 at 37°C for 1 h. Between steps, the membrane was washed three times with PBS 5 minutes. For Dot blot assays, the samples were vacumm blotted onto a nitrocellulose membrane and treated as mentioned for Western blot.

### VLPs purification by sucrose cushion ultracentrifugation

Supernatants of previously transfected cells were collected and clarified from cellular debris by low speed centrifugation at 2800 g, at room temperature for 20 min. After clarification, VLPs present in supernatants were pelleted through a 30% (w/v) sucrose cushion at 96,000 g for 2 h at 4°C. Pellets were then resuspended in PBS. Corresponding monolayers were harvested in ProteoJET Mammalian Cell Lysis Reagent (Fermentas) and used as control samples in Western blot assays.

In order to obtain large quantities of Z-eGFP VLPs, multiple 75 cm^2^ culture flasks at 80% confluence were transfected with pZ-eGFP and culture media was collected up to the third passage. Following the first step of ultracentrifugation the pellets were pooled and resuspended on PBS for further treatment with proteinase K. An extra ultracentrifugation step (96000 g for 2 h at 4°C) was made for the removal of the proteinase K, foreign proteins and defective VLPs from the preparation. The pellet was resuspended in PBS and used to immunize mice.

### Protease protection assay

Pellets obtained from pZ-eGFP transfected cells were treated with 0.4 μg, 0.08 μg or 0.04 μg of proteinase K (Fermentas) alone or with a combination of proteinase K and 1% Triton X-100 for 30 min at 37°C. After the incubation the reaction was stopped with 100 mM PMSF and boiled for 10 minutes. Samples were then analyzed by SDS-PAGE and Western Blot.

### Transmission electron microscopy (TEM)

A drop of the purified VLPs suspension was deposited on a formvar-carbon-coated nickel grid for 1 min. The samples were negatively stained with 2% phosphotungstic acid and examined in a Phillips EM 301 electron microscope.

### TEM-immunogold

After ultracentrifugation, VLPs were resuspended in PBS containing 4% paraformaldehyde. A drop of the suspension was deposited on a formvar-carbon-coated nickel grid for 1 min and washed six times with PBS. The grids were then treated with dilution buffer i.e. PBS containing 0.5% bovine serum albumin and 0.1% Tween during 30 min followed by washing with PBS. Grids were then incubated for 1 h with rabbit anti-Z polyclonal IgG 1/300 in dilution buffer for 1 hour. The grids were washed six times with PBS and incubated further for 1 h with a donkey anti-rabbit IgG antibody coupled to 12 nm gold beads (Invitrogen) 1/20 in dilution buffer. Finally, grids were washed six times with PBS, fixed for 10 min in 2% glutaraldehyde, negatively stained with uranil acetate and examined by TEM.

### Mice immunization

Male inbred Balb/C mice aged 6 weeks (4 per group) were immunized intramuscularly with 5 μg of Z-eGFP VLPs in 100 μl PBS, twice threat a three weeks interval. To determine the effect of VLP integrity on its inmunogenicity, VLPs were disrupted by treatment with 1% Triton X-100 and used to immunize mice as a control group. Two additional controls groups were included: mice immunized with either 5 μg of soluble GFP protein or PBS alone. Blood samples were collected on days 0, 7, 14, 21, 28 and 35 for analysis of immune responses and mice were sacrified on day 42 postimmunization. Sera were stored at −20°C until use.

### Evaluation of humoral immune response

Ninety six-well microtiter plates (Nunc, Maxisorpt Glostrup, Denmark) were coated with 0.5 μg of GFP protein in coating buffer, ie: 0.1 M bicarbonate buffer pH 9.6, overnight at 4°C. Plates were blocked with PBS containing 1% of casein at 37°C, for 1 h. After this and the following steps the plates were washed three times with washing solution, i.e.: PBS, 0.5 M NaCl, 0.2% (v/v) Triton X-100. Fifty microliters of each serum sample from immunized animals diluted in sample buffer (i.e.: 1% (w/v) casein in washing solution) were added to the antigen coated wells and incubated at 37ºC for 1 h. Bound antibodies were detected with peroxidase-conjugated anti-mouse IgG (Pierce) in sample buffer at 37°C for 1 h and were then revealed with ortho-phenylene diamine (OPD, Sigma Chemicals Co) [42].

## Abbreviations

CMV: Cytomegalovirus; MVB: Multivesicular bodies; pDC: Plasmacytoid dendritic cells; PML: Promyelocytic Leukemia Protein; TEM: Transmission electron microscopy; VLP: Virus-like-particles.

## Competing interest

The authors declare that they have no competing interests.

## Authors’ contributions

CSB carried out all the experiments and drafted the manuscript; MFB design the molecular experiments and participated in the general direction; MHA designed the Immunological tests and helped with the analysis and interpretation of data; SEG participated in the design and realization of the manuscript; JAI collaborated with the preparation of materials needed for all assays; GG participated in the analysis and drafting of the manuscript; MEL conceived the work and coordinated the drafting of the manuscript. All authors read and approved the final manuscript.

## Authors’ information

CSB, MFB, MHA, SEG, JAI, GG, and MEL are research-professors of the Universidad Nacional de Quilmes. CSB holds a doctoral fellowship of Consejo Nacional de Ciencia y Tecnología (CONICET). MFB is member of the Research Career of CONICET. SEG holds a postdoctoral fellowship of CONICET. JAI is professor of the Universidad Nacional Arturo Jauretche. MEL is member of the Research Career of CONICET.

## Supplementary Material

Additional file 1 **Figure S1.** Individual optical sections of transfected 293T cells. A. Optical sections of pZ-eGFP transfected cells, from section 21–67. B. Optical sections of control peGFP transfected cells, from section 16–61. The thickness of the optical sections is 3.9 nm. Click here for file

Additional file 2 **Figure S2.** Transmission electron microscopy with immunogold labeling. Transmission electron microscopy with immunogold labeling of the purified VLPs by ultracentrifugation through a sucrose cushion. Z-EGFP VLPs are pointed with red arrows, and similar structures that are not immunogold labeled are pointed with empty arrows. (The VLP indicated with * is amplified in Figure 2C). The bar represents 100 nm. Click here for file
