# Supplementary figures and images for: Antigen vehiculization particles based on the Z protein of Junin virus
Source: BMC Biotechnol. 2012 Nov 2;12:80. doi: 10.1186/1472-6750-12-80 (PMC3534497; doi:10.1186/1472-6750-12-80)

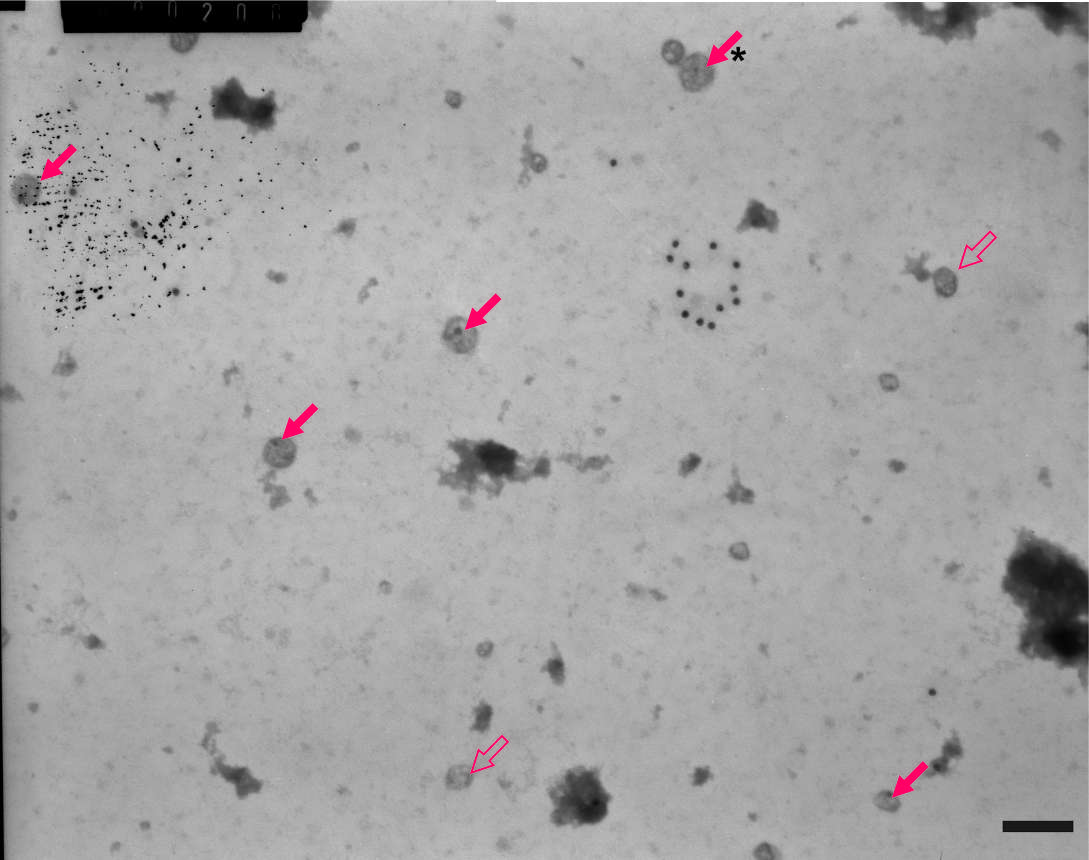

Supplement: Additional file 2 — Figure S2. Transmission electron microscopy with immunogold labeling. Transmission electron microscopy with immunogold labeling of the purified VLPs by ultracentrifugation through a sucrose cushion. Z-EGFP VLPs are pointed with red arrows, and similar structures that are not immunogold labeled are pointed with empty arrows. (The VLP indicated with * is amplified in Figure 2C). The bar represents 100 nm. [file 1472-6750-12-80-S2.jpeg]
